# Detection of *Mycobacterium leprae* DNA in soil: multiple needles in the haystack

**DOI:** 10.1038/s41598-019-39746-6

**Published:** 2019-02-28

**Authors:** Maria Tió-Coma, Thomas Wijnands, Louise Pierneef, Anna Katarina Schilling, Korshed Alam, Johan Chandra Roy, William R. Faber, Henk Menke, Toine Pieters, Karen Stevenson, Jan Hendrik Richardus, Annemieke Geluk

**Affiliations:** 1 Department of Infectious Diseases, Leiden University Medical Center, Leiden, The Netherlands; 2 Royal (Dick) School of Veterinary Studies and Roslin Institute, University of Edinburgh, Roslin, Scotland United Kingdom; 3 Rural Health Program, The Leprosy Mission International Bangladesh, Nilphamari, Bangladesh; 4 Department of Dermatology, Amsterdam UMC, University of, Amsterdam, The Netherlands; 5 Division Pharmacoepidemiology and Clinical Pharmacology, Utrecht Institute for Pharmaceutical Sciences, Utrecht, The Netherlands; 6 Moredun Research Institute, Pentlands Science Park, Edinburgh, Scotland United Kingdom; 7 Department of Public Health, Erasmus MC, University Medical Center Rotterdam, Rotterdam, The Netherlands

## Abstract

Leprosy is an infectious disease caused by *Mycobacterium leprae* affecting the skin and nerves. Despite decades of availability of adequate treatment, transmission is unabated and transmission routes are not completely understood. Despite the general assumption that untreated *M. leprae* infected humans represent the major source of transmission, scarce reports indicate that environmental sources could also play a role as a reservoir. We investigated whether *M. leprae* DNA is present in soil of regions where leprosy is endemic or areas with possible animal reservoirs (armadillos and red squirrels). Soil samples (n = 73) were collected in Bangladesh, Suriname and the British Isles. Presence of *M. leprae* DNA was determined by RLEP PCR and genotypes were further identified by Sanger sequencing. *M. leprae* DNA was identified in 16.0% of soil from houses of leprosy patients (Bangladesh), in 10.7% from armadillos’ holes (Suriname) and in 5% from the habitat of lepromatous red squirrels (British Isles). Genotype 1 was found in Bangladesh whilst in Suriname the genotype was 1 or 2. *M. leprae* DNA can be detected in soil near human and animal sources, suggesting that environmental sources represent (temporary) reservoirs for *M. leprae*.

## Introduction

Leprosy is a debilitating infectious disease caused by *Mycobacterium leprae* and *Mycobacterium lepromatosis* that is still considered a major threat in developing countries by WHO, remaining persistently endemic in regions in Africa, South America and Asia. Every year more than 200,000 new patients are still diagnosed and this new case detection rate has been virtually stable over the last decade^1^. These facts indicate that multidrug therapy (MDT), although effective to treat leprosy, is insufficient to prevent transmission^2^.

Granting *M. leprae* transmission is not completely understood, risk factors for development of leprosy have been identified including close contact with untreated, multibacillary patients^3^, human susceptibility genes^4,5^, infection with soil transmitted helminths^6^, as well as food shortage^7^.

The mechanism by which bacteria are transmitted from one organism to another has not been unequivocally demonstrated^8^. However, based on existing evidence, skin-to-skin contact, aerosols as well as shedding of bacteria into the environment subsequently followed by infection of other individuals remain the most obvious options for human leprosy^8,9^. Still these routes provide no explanation for the occurrence of leprosy in individuals without known contact to leprosy patients or in areas without any reported new cases^9,10^.

Through PCR amplification of *M*. *leprae* DNA, its presence has been detected in environmental samples such as soil^11–16^ and water^17,18^ in areas inhabited by leprosy patients in Brazil and India. The viability of *M*. *leprae* was assessed by its multiplication in footpads of wild type mice and showed that *M*. *leprae* can remain alive in wet soil for 46 days^19^. Moreover, viability of *M*. *leprae* bacilli in soil from India has been studied by 16S ribosomal RNA gene analysis^20^. This study showed that 25% of the soil samples collected from patients’ areas contained *M*. *leprae* 16S ribosomal RNA, suggesting the presence of viable *M*. *leprae* in the soil. Additionally, if environment–free living amoebic cysts cultured in the laboratory are artificially infected with *M*. *leprae* (bacilli:amoebae ratio of 5–10:1), the bacteria can survive up to 8 months^21^.

Recently, *M*. *leprae* and *M*. *lepromatosis* were identified in red squirrels from the British Isles causing lepromatous disease in several animals^22,23^. Phylogenetic analyses determined that the *M*. *leprae* strain in squirrels (3I) was related to the lineage circulating in Medieval England, suggesting the red squirrels as a contemporary reservoir of the bacilli.

Zoonotic transmission of *M*. *leprae* from armadillos has been detected in the southeastern United States where wild armadillos and patients were infected with the same genotype (3I-2-v1)^24^.

Furthermore, although the prevalence of leprosy in nonhuman primates (NHP) seems to be quite low, *M*. *leprae* infections have also been reported in NHP^25^ carrying *M*. *leprae* strains closely related to the human strains, suggesting that NHPs transmission can occur from human (or human sources like trash), but also among NHPs^25^.

In this study, we aimed to explore whether besides humans and animals, environmental sources may function as a reservoir of *M*. *leprae*. For this purpose, we investigated the presence of *M*. *leprae* DNA in soil from regions with varying human leprosy endemicity in Bangladesh, Suriname, Brownsea Island and the Isle of Arran^22^.

## Materials and Methods

### DNA extraction from soil

Moist soil samples from 3 regions (Supplementary Table 1) were collected at a depth of 2 cm (Bangladesh and Suriname) or 8 cm (British Isles) in areas without sun light and stored in 50 ml tubes (Greiner Bio-One, Kremsmünster, Austria): i) in Bangladesh in front of the bedroom (right on the doorstep) in the houses of leprosy patients (n = 25) and from areas without known leprosy patients (n = 2); ii) in Suriname (Batavia and Groot Chatillon (former leprosy colonies), Pikin Slee and Gujaba) from areas known to be inhabited by nine-banded armadillos (n = 28) (samples Suriname 2, 3 and 6 from Batavia and Groot Chatillon were previously described (van Dissel *et al*. submitted) and are presented here for reference purposes); iii) in the British Isles in the habitat of Eurasian red squirrels carrying *M*. *leprae* (Brownsea Island, n = 10) and *M*. *lepromatosis* (Isle of Arran, n = 10).

As a negative control soil was obtained from the surroundings of the Leiden University Medical Centre (The Netherlands) and spiked with 10^8^ cells of *M*. *leprae* NHPD-63 as positive control.

DNA was extracted from 10 g of soil using DNeasy PowerMax Soil (Qiagen, Valencia, CA) as per manufacturer’s instructions.

### PCR amplification of RLEP and LPM244

To detect the presence of *M*. *leprae* DNA in soil, a PCR amplifying an *M*. *leprae*-specific repetitive sequence (RLEP) was performed. PCR amplification of a 129 bp sequence of RLEP^26^ was carried by addition of 10 µl 5x Gotaq® Flexi buffer (Promega, Madison, WI), 5 µl MgCl_2_ (25 mM), 2 µl dNTP mix (5 mM), 0.25 µl Gotaq® G2 Flexi DNA Polymerase (5 u/µl), 5 µl (2 µM) forward and reverse primers (Supplementary Table 2) and 5 µl template DNA in a final volume of 50 µl. DNA from *M*. *bovis* BCG P3 and *M*. *tuberculosis* H37Rv were used to assess PCR-specificity. As PCR positive controls DNA from *M*. *leprae* Br4923 and Thai-53 were used.

To detect inhibition of PCR due to remaining soil components, 1 µl of *M*. *leprae* DNA was added to the aforementioned PCR mixes together with 5 µl template DNA. In samples presenting PCR inhibition, 5 µl (2 mM) Bovine Serum Albumin (BSA) Fraction V (Roche Diagnostics, Indianapolis, IN) were added to the PCR mixes.

PCR mixes were denatured for 2 min at 95 °C followed by 40 cycles of 30 s at 95 °C, 30 s at 65 °C and 30 s at 72 °C and a final extension of 10 min at 72 °C. PCR products (15 µl) were used for electrophoresis in a 3.5% agarose gel at 130 V. Amplified DNA was visualized by Midori Green Advance staining (Nippon Genetics Europe, Dueren, Germany) using a Gel Doc System (Bio-Rad Laboratories, Hercules, CA).

PCR to detect *M*. *lepromatosis* was performed for soil from the British Isles. The primers (LPM244) amplify a 244 bp region of the *hemN* gene not present in *M*. *leprae* or other mycobacteria^27^. PCR was performed as explained above with LMP244 primers (Supplementary Table 2) and an annealing temperature of 53 °C. *M*. *lepromatosis* DNA was used as a positive control.

### Genotyping

To determine the genotype (1, 2, 3 or 4) of *M*. *leprae*, SNP-14676 (locus 1), SNP-1642875 (locus 2) and SNP-2935685 (locus 3) were amplified and sequenced as described^28^ with minor modifications: PCRs were performed with 5 µl of template DNA using the aforementioned PCR mixes and forward and reverse primers for loci 1–3 (Supplementary Table 2) in a final volume of 50 µl. DNA was denatured for 2 minutes at 95 °C, following 45 cycles of 30 s at 95 °C, 30 s at 58 °C and 30 s at 72 °C and a final extension cycle of 10 min at 72 °C. PCR products were resolved by agarose gel electrophoresis as explained above. PCR products showing a band were purified prior to sequencing using the Wizard SV Gel and PCR Clean-Up System (Promega, Madison, WI). Sequencing was performed on the ABI3730xl system (Applied Biosystems, Foster City, CA) using the BigDye Terminator Cycle Sequencing Kit (Thermo Fisher Scientific, Waltham, MA).

## Results

### Detection of *M*. *leprae* DNA in soil

To determine whether *M*. *leprae* DNA is present in the environment surrounding leprosy patients, the habitat of armadillos and red squirrels with leprosy-like disease, soil was collected in each area. PCR amplification of a 129 bp sequence of the RLEP region from *M*. *leprae* was performed in a total of 75 soil samples from 3 different regions (Supplementary Table 1). Control soil samples did not show amplification of the fragment in RLEP PCR, whereas the same sample spiked with *M*. *leprae* bacilli presented a clear band confirming the applicability of the method to isolate, purify and detect *M*. *leprae* in soil. PCR amplification of 5 µl of *M*. *bovis* BCG P3 and *M*. *tuberculosis* H37Rv DNA did not show amplification of RLEP showing specificity of the PCR for *M*. *leprae* DNA.

**Figure 1:**
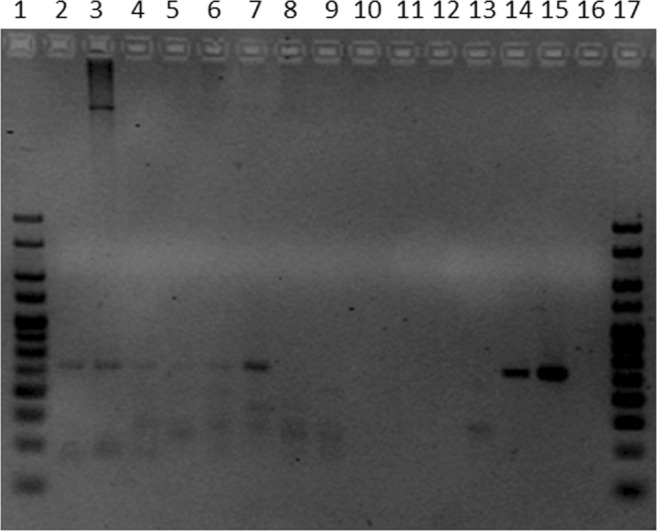
Gel of PCR for RLEP region to detect presence of *M. leprae* in soil samples. PCR products were electrophoresed in a 3.5% agarose gel. The size of the amplified RLEP sequence is 129 bp. Lanes 2 to 4 represent soil samples collected in Suriname (Suriname 2, 3, and 6), lanes 5 to 14 are soil samples collected in Bangladesh (01/65959/00, 01/65922/00, 01/65958/00, 02/65971/00, 02/22705/00, 01/65945/00, 01/65942/00, 01/65975/00, 01/22711/00 and 01/22723/00), lane 15 is DNA of *M*. *leprae* Thai-53 strain, lane 16 is a negative PCR control and lanes 1 and 17 are 25 bp HyperLadder (Bioline, Taunton, MA).

**Figure 2:**
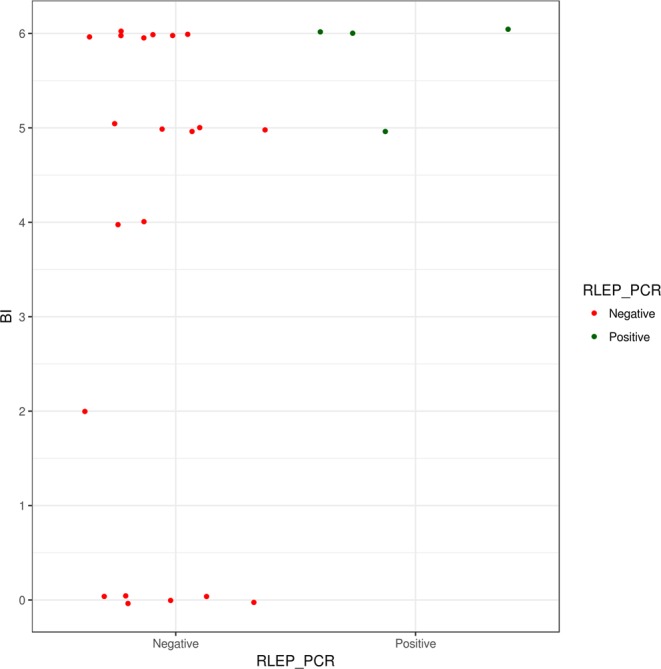
RLEP PCR positivity in soil samples from Bangladesh and bacillary load (BI) of patient. Soil samples collected in Bangladesh are represented in the graph by dots and sorted based on RLEP PCR results and bacillary load of the patient living in the household where the soil was collected.

In Bangladesh, 4 out of 25 collected samples were positive for RLEP PCR (Fig. 1, Table 1; Supplementary Table 3), all of which were collected in houses of leprosy patients with high bacillary load (BI 5–6, Fig. 2). *M*. *leprae* DNA was not detected in the two soil samples from areas in Bangladesh without any reported leprosy cases (Supplementary Fig. 1).

**Table 1:** RLEP PCR results for *M. leprae* DNA derived from soil samples.

Origin	Positive		Negative	
	Number	%	Number	%
Bangladesh	4	16.0	21	84.0
Suriname	3	10.7	25	89.3
Brownsea Island	1	10.0	9	90.0
Isle of Arran	0	0.0	10	100.0

RLEP PCR result to detect *M*. *leprae* DNA in soil samples from Bangladesh, Suriname, Brownsea Island and Isle of Arran. A positive result is determined by a visible band of 129 bp in an agarose gel.

In Suriname, samples (n = 28) were taken in three different locations inhabited by armadillos and *M*. *leprae* DNA was detected in 3 samples obtained at former leprosy colonies in Batavia and Groot-Chatillon (Fig. 1, Table 1; Supplementary Table 4).

Since all PCRs performed with UK samples were negative, we investigated whether PCRs were inhibited by compounds in the soil. DNA of *M*. *leprae* was added to the PCR mixes containing the DNA isolated from all soil samples and inhibition of PCR was determined by a negative PCR result. Inhibition was observed in 7 of the 10 soil samples from Brownsea Island, 8 out of the 10 from the Isle of Arran and 1 out of the 28 from Suriname. Since humic acid in soil can act as a PCR inhibitor^29,30^, 5 µl of 2 mM BSA was added to the PCRs with soil samples from the British Isles to overcome inhibition. Indeed, addition of BSA to soil-DNA spiked with *M*. *leprae* DNA (Br4923 or Thai-53), resulted in PCR-positivity for all spiked samples, indicating that BSA can prevent PCR inhibition due to undetermined soil compounds (data not shown).

Ten soil samples were collected in the surroundings of the infected red squirrels one of which was RLEP PCR positive (Tables 1 and 2). To determine whether *M*. *lepromatosis* DNA was also present in soil from the Isle of Arran with reported *M*. *lepromatosis* infection in red squirrels, PCRs were performed amplifying a 244 bp region of the *hemN* gene unique of *M*. *lepromatosis*^27^. None of the 10 soil samples collected resulted in PCR-positivity using LPM244 primers.

**Table 2:** SNP typing results.

	Locus 1	Locus 2	Locus 3	Genotype
Tamil Nadu (reference strain)	C	G	A	1
Br4923 (reference strain)	T	T	C	4
Suriname 2	UD	UD	A	1 or 2
Suriname 3	UD	UD	A	1 or 2
Suriname 6	C	UD	A	1 or 2
Bangladesh 01/65922/00	UD	G	UD	1
Bangladesh 01/65958/00	UD	G	UD	1
Bangladesh 01/22723/00	C	G	A	1

Polymorphic sites in the genome of *M*. *leprae*: locus 1 (SNP-14676), locus 2 (SNP-1642875) and locus 3 (SNP-2935685) and the corresponding genotype. Nucleic acid corresponding to each polymorphic site of *M*. *leprae* reference strains Tamil Nadu and Br4923 and soil samples that were successfully sequenced. When PCR amplification or sequencing of the locus was not successful it is marked as undetermined (UD).

Next, for all RLEP PCR positive samples from Bangladesh (n = 4), Suriname (n = 3) and the British Isles (n = 1) the PCR-amplified 129 bp RLEP region was sequenced. Sequence alignment with the RLEP region of *M*. *leprae* was found for all 8 samples, confirming that *M*. *leprae* specific DNA can be identified in soil using the above described procedure.

### Genotyping

**Figure 3:**
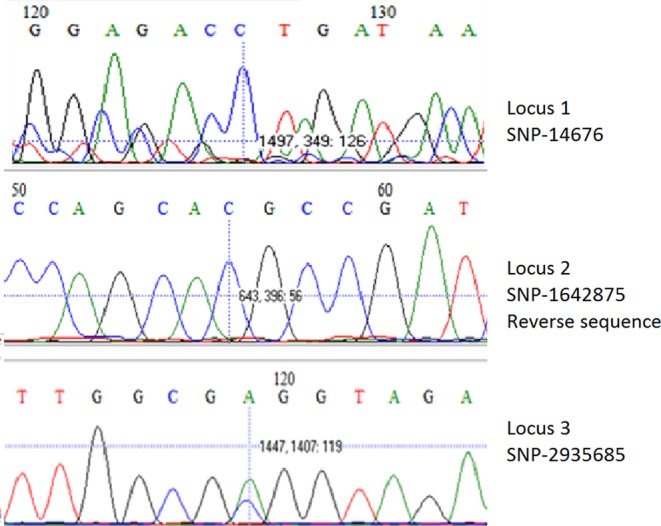
SNP analysis of loci 1, 2 and 3 from a representative *M*. *leprae* positive soil sample collected in Bangladesh. Sequencing results of locus 1 (SNP-146763) top, reverse sequence of locus 2 (SNP-1642875) middle and locus 3 (SNP-2935685) bottom, from soil sample Bangladesh 01/22723/00 used to determine the genotype of the *M*. *leprae* strain identified (genotype 1). SNP positions are based on the *M*. *leprae* TN strain. Vertical bars indicate the polymorphic base.

Genotypes of the RLEP PCR positive soil samples (n = 8) were investigated and determined according to the combination of SNPs in loci 1–3 as described by Monot *et al*.^28^ RLEP-positive soil from Bangladesh were classified as genotype 1 (Table 2) according to the polymorphism in locus 2 or loci 1–3 (01/22723/00, Fig. 3). For the soil from Suriname the genotype was narrowed down to either 1 or 2 since only sequencing of locus 3 (Suriname 2, 3 and 6) and locus 1 (Suriname 6) were identified. For the RLEP positive sample from Brownsea Island it was not possible to obtain sequence information for any of the polymorphic loci to assign a genotype. This was most likely due to the small amount of *M*. *leprae* DNA in the samples.

## Discussion

Human leprosy still poses a considerable health threat in developing countries where transmission is generally assumed to take place via aerosol droplets from nasal cavities of untreated *M*. *leprae* infected individuals to their close contacts^8,9^. However, nonhuman animal and environmental sources have also been suggested to play a role in the pathogen’s dissemination^9^. As paleopathological evidence of leprosy in pre-Columbian America is lacking, leprosy was very likely introduced to the continent by European colonists or the African slave route^28^ also resulting in transmission to armadillos. However, nowadays infected armadillos may even be responsible for new cases in human individuals who have never had contact with leprosy patients nor travelled to leprosy endemic areas^10,31^. In addition, another living host that could potentially represent an environmental reservoir for *M*. *leprae* are amoebae as it has been shown that *M*. *leprae* can survive in free living amoebae^21^. Thus, amoebas or other protists might represent an intermediate host which would allow indirect infection with *M*. *leprae* through environmental samples.

In this study, *M*. *leprae* DNA was identified in soil collected in the houses of leprosy patients and the habitats of armadillos and red squirrels, suggesting that soil may represent a (temporary) reservoir. However, this study did not asses viability of the bacteria and since *M*. *leprae* is an obligate intracellular pathogen further investigation is needed to elucidate the role of the environment in *M*. *leprae* transmission.

Understanding how *M*. *leprae* is transmitted, and identifying sources of infection is crucial to prevent new cases and thus blocking transmission is essential to ultimately eradicate leprosy.

Although human leprosy was eradicated from the British Isles centuries ago, Eurasian red squirrels have remained a reservoir for *M*. *leprae*, containing a strain closely related to the strain present in Medieval England (3I). This indicates that *M*. *leprae* may have persisted in the environment after the human reservoir disappeared. However, *M*. *leprae* DNA was not abundantly present in soil, suggesting that the risk of environmental contamination is low.

Because the genome of *M*. *lepromatosis* contains only one copy of the *hemN* gene^32^ detected by LPM244 whereas 37 copies^33^ are present in the RLEP region^34^ of *M*. *leprae*, an equal amount of bacteria would be less well detectable by LPM244 PCR for *M*. *lepromatosis* than by RLEP PCR for *M*. *leprae*. Added to the fact that *M*. *lepromatosis* prevalence in the squirrel population is low, it is therefore possible that sensitivity was not sufficient to detect *M*. *lepromatosis*.

In Bangladesh, *M*. *leprae* was only found in soil collected in the houses of patients with high BI index (Fig. 2). At those locations more bacteria are shed and thus the likelihood of encountering bacteria in the soil is higher. However, a high BI index of the patient where the soil sample was collected was not necessarily associated with a positive RLEP PCR result. The higher percentage of RLEP positive soil in Bangladesh is likely due to a more targeted selection of the sample location in the houses of leprosy patients as well as the higher leprosy prevalence.

In previous phylogeographic analysis genotype 1 was identified as the predominant strain type in South Asia^35,36^ and was likely introduced to South Asia from other parts of that continent^36^. The genotype found in soil samples from Bangladesh (1) is therefore in accordance with previous phylogeographic data^35^.

In summary, this study demonstrates the presence of *M*. *leprae* DNA in soil, contributing to a OneHealth view on transmission including humans, animals and the environment. Further research is needed, however, to confirm whether *M*. *leprae* DNA in soil is derived from viable bacteria that can survive in smaller hosts such as helminths or amoebas. Thus, strategies aimed at prevention of transmission by administration of post-exposure prophylaxis to infected individuals should, besides human reservoirs of *M*. *leprae*, also consider environmental sources of (re)infection.

## Supplementary information

Supplementary materials
