# Supplementary material for: Detection of Mycobacterium leprae DNA in soil: multiple needles in the haystack
Source: Sci Rep. 2019 Feb 28;9:3165. doi: 10.1038/s41598-019-39746-6 (PMC6395756; doi:10.1038/s41598-019-39746-6)
Supplement: Supplementary file 1 — Supplementary materials [file 41598_2019_39746_MOESM1_ESM.docx]

**Detection of *Mycobacterium leprae* DNA in soil: multiple needles in the haystack**

**Maria Tió-Coma^1^, Thomas Wijnands^1^, Louise Pierneef^1^, Anna Katarina Schilling^2^, Korshed Alam^3^, Johan Chandra Roy^3^, William R. Faber^4^, Henk Menke^5^, Toine Pieters^5^, Karen Stevenson^6^, Jan Hendrik Richardus*^7,^* and Annemieke Geluk^1*^**

*From the ^1^Department of Infectious Diseases, Leiden University Medical Center, Leiden, The Netherlands;* ***^2^****Royal (Dick) School of Veterinary Studies and Roslin Institute, University of Edinburgh, Roslin, Scotland, United Kingdom;* ***^3^****Rural Health Program, The Leprosy Mission International Bangladesh, Nilphamari, Bangladesh; ^4^Department of Dermatology, Amsterdam UMC, University of Amsterdam, The Netherlands; ^5^Division Pharmacoepidemiology and Clinical Pharmacology, Utrecht Institute for Pharmaceutical Sciences, Utrecht, The Netherlands; and from the;* ^6^*Moredun Research Institute, Pentlands Science Park, Edinburgh, Scotland, United Kingdom;* *^7^Department of Public Health, Erasmus MC, University Medical Center Rotterdam, Rotterdam, The Netherlands.*

# Supplementary Tables

**Supplementary Table 1***.* ***Origin, number and location of soil samples.***

| **Origin** | **Number** | **Area of collection** |
| --- | --- | --- |
| Bangladesh | 25 | Houses of leprosy patients |
| Bangladesh | 2 | Area without any reported case of leprosy |
| Suriname | 28 | Surroundings of armadillos’ habitats |
| British Isles | 20 | Areas frequented by red squirrels infected with *M. leprae* (Brownsea Island) or *M. lepromatosis* (Arran Isle) |
| The Netherlands | 2 | Control soil |

Summary of soil collected and brief description of area.

**Supplementary Table 2**. ***PCR primers for RLEP region and loci 1, 2 and 3 of M. leprae.***

| **Primer** | **Sequence 5** '**-3** **'** | **Fragment Size (bp)** | **Reference** |
| --- | --- | --- | --- |
| RLEP LP1 F | TGCATGTCATGGCCTTGAGG | 129 | 21 |
| RLEP LP2 R | CACCGATACCAGCGGCAGAA |  |  |
| LPM244 F | GTTCCTCCACCGACAAACAC | 244 | 22 |
| LPM244 R | TTCGTGAGGTACCGGTGAAA |  |  |
| Locus 1 F (SNP 14676) | AATGGAATGCTGGTGAGAGC | 148 | 23 |
| Locus 1 R (SNP 14676) | CAATGCATGCTAGCCTTAATGA |  |  |
| Locus 2 F (SNP 1642875) | TTGAATGCGACCAAACGTACTTTCTG | 114 | 29 |
| Locus 2 R (SNP 1642875) | TACCACCGGATCATGGAACCGTC |  |  |
| Locus 3 F (SNP 2935685) | ATCTGGTCCGGGTAGGAATC | 180 | 23 |
| Locus 3 R (SNP 2935685) | ACCGGTGAGCGCACTAAG |  |  |

Primers for loci 1, 2 and 3 used for PCR and sequencing.

**Supplementary Table 3. *Location, PCR results and genotype of M. leprae of soil samples from Bangladesh.***

| **Sample ID** | **GIS coordinates** | **BI of index case** | **RLEP PCR** | **Genotype** |
| --- | --- | --- | --- | --- |
| 01/65938/00 | N25.92199º  E88.94093 | 6 | Negative |  |
| 01/22680/00 | N25.96251  E88.25130 | 2 | Negative |  |
| 01/65939/00 | N25.88011  E89.03565 | 5 | Negative |  |
| 01/65959/00 | N25.33035  E89.21510 | 5 | **Positive** | ND |
| 01/65922/00 | N26.10478  E88.54177 | 6 | **Positive** | 1 |
| 01/65930/00 | N25.99863  E88.86437 | 5 | Negative |  |
| 01/65958/00 | N25.45427  E89.13233 | 6 | **Positive** | 1 |
| 02/65971/00 | N25.37141  E89.07424 | 0 | Negative |  |
| 02/22705/00 | N26.18514  E88.31788 | 0 | Negative |  |
| 02/65968/00 | N25.60300  E89.02471 | 0 | Negative |  |
| 02/65970/00 | N25.91928  E89.13503 | 0 | Negative |  |
| 02/65956/00 | N25.92107  E88.92709 | 0 | Negative |  |
| 02/22707/00 | N26.05023  E88.35850 | 0 | Negative |  |
| 01/65945/00 | N26.06099  E88.49534 | 6 | Negative |  |
| 01/65942/00 | N25.90073  E89.02189 | 4 | Negative |  |
| 01/65975/00 | N26.05229  E88.93260 | 5 | Negative |  |
| 01/22711/00 | N25.72158  E88.39340 | 4 | Negative |  |
| 01/22723/00 | N25.99967  E88.33660 | 6 | **Positive** | 1 |
| 01/22726/00 | N25.96332  E88.32334 | 6 | Negative |  |
| 01/66210/00 | N25.49954  E89.06985 | 6 | Negative |  |
| 01/66105/00 | N26.08686  E88.56291 | 5 | Negative |  |
| 01/66058/00 | N25.50946  E89.15213 | 6 | Negative |  |
| 01/66084/00 | N25.80686  E88.87104 | 5 | Negative |  |
| 01/66003/00 | N26.11445  E88.55625 | 6 | Negative |  |
| 01/66156/00 | N26.16790  E88.46975 | 6 | Negative |  |
| Control group Kacharipara | N26.01995  E88.82793 | NA | Negative |  |
| Control group Hartakitola | N26.00494  E88.83931 | NA | Negative |  |

**ND** indicates that the genotype could not be determined due to no amplification of the PCR for loci 1-3.

**Supplementary Table 4. *Location, PCR results and genotype of M. leprae of soil samples from Suriname .***

| **Sample ID** | **GIS coordinates** | **Area** | **RLEP PCR** | **Genotype** |
| --- | --- | --- | --- | --- |
| Suriname 1 | N5.413004  W55.52597 | Batavia | Negative |  |
| Suriname 2 | N5.413004 W55.52597 | Batavia | **Positive** | 1 or 2 |
| Suriname 3 | N5.413194  W55.52564 | Batavia | **Positive** | 1 or 2 |
| Suriname 4 | N5.413194  W55.52564 | Batavia | Negative |  |
| Suriname 5 | N5.413194  W55.52564 | Batavia | Negative |  |
| Suriname 6 | N5.37203  W55.42430 | Groot Chatillon | **Positive** | 1 or 2 |
| Suriname 7 | N5.37203  W55.42430 | Groot Chatillon | Negative |  |
| Suriname 2018/01 | N4.14376  W55.26411 | Pikin Slee | Negative |  |
| Suriname 2018/02 | N4.14776  W55.26411 | Pikin Slee | Negative |  |
| Suriname 2018/03 | N4.14776  W55.26411 | Pikin Slee | Negative |  |
| Suriname 2018/04 | N4.14771  W55.26391 | Pikin Slee | Negative |  |
| Suriname 2018/05 | N4.14771  W55.26391 | Pikin Slee | Negative |  |
| Suriname 2018/06 | N4.14771  W55.26391 | Pikin Slee | Negative |  |
| Suriname 2018/07 | N4.18762  W55.24725 | Pikin Slee | Negative |  |
| Suriname 2018/08 | N4.18759  W55.24730 | Pikin Slee | Negative |  |
| Suriname 2018/09 | N4.18602  W55.24986 | Goejaba | Negative |  |
| Suriname 2018/10 | N4.18589  W55.25056 | Goejaba | Negative |  |
| Suriname 2018/11 | N4.18579  W55.25003 | Goejaba | Negative |  |
| Suriname 2018/13 | N4.15554  W55.27434 | Pikin Slee | Negative |  |
| Suriname 2018/14 | N4.15870  W55.27687 | Pikin Slee | Negative |  |
| Suriname 2018/15 | N4.13217  W55.26315 | Pikin Slee | Negative |  |
| Suriname 2018/16 | N4.18480  W55.24330 | Goejaba | Negative |  |
| Suriname 2018/17 | N4.18400  W55.25060 | Goejaba | Negative |  |
| Suriname 2018/18 | N4.18341  W55.24592 | Goejaba | Negative |  |
| Suriname 2018/19 | N4.18413  W55.24595 | Goejaba | Negative |  |
| Suriname 2018/20 | N4.14471  W55.26314 | Pikin Slee | Negative |  |
| Suriname 2018/21 | N4.14802  W55.26521 | Pikin Slee | Negative |  |
| Suriname 2018/22 | N4.14855  W55.26510 | Pikin Slee | Negative |  |

# Supplementary figures

**
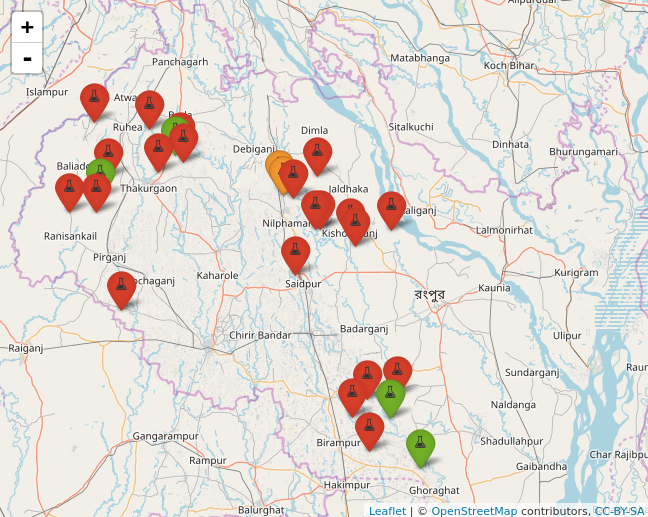
 Supplementary Fig 1**. **Soil sampling sites in Bangladesh.**

Red markers indicate a negative result for RLEP PCR. Green markers indicate presence of *M. leprae* determined by a positive result for RLEP PCR. Orange markers illustrate the soil collected in areas not known to be inhabited by leprosy patients. Both soil samples collected in areas not known to be inhabited by leprosy patients were negative for RLEP PCR. The figure was drawn in R (v3.4.3) with the package *leaflet* (v1.1.0.9000) using maps available under the Open Database License from © OpenStreetMap contributors (https://www.openstreetmap.org/copyright).


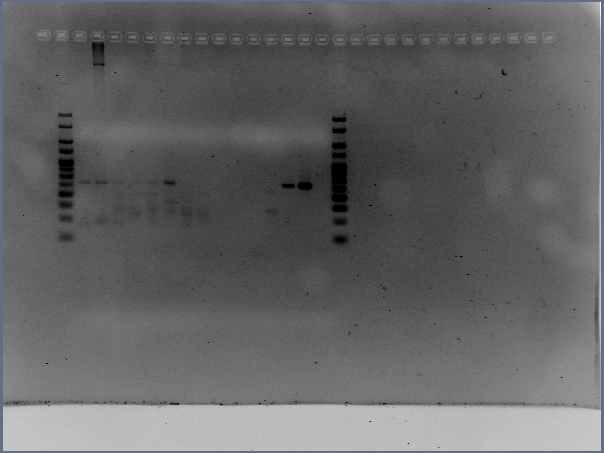
 **Supplementary Fig 2**. **Full length gel shown in Figure 1.**

Picture is cropped and numbers are introduce in Figure 1 for easier interpretation.
